# Non-pharmaceutical Interventions and the Infodemic on Twitter: Lessons Learned from Italy during the Covid-19 Pandemic

**DOI:** 10.1007/s10916-021-01726-7

**Published:** 2021-03-06

**Authors:** Maurizio Massaro, Paola Tamburro, Matteo La Torre, Francesca Dal Mas, Ronald Thomas, Lorenzo Cobianchi, Paul Barach

**Affiliations:** 1 Dipartimento di Management, Università Ca’ Foscari, Venice, Italy; 2 Freelance Data Scientist, Rome, Italy; 3 Dipartimento di Economia, Università G. d’Annunzio, Chieti-Pescara, Italy; 4 Ipazia Observatory on Gender Research, Rome, Italy; 5 Lincoln International Business School, University of Lincoln, Lincoln, UK; 6 Department of Pediatrics, School of Medicine, Central Michigan University, Mt Pleasant, MI USA; 7 Dipartimento di Scienze Clinico-Chirurgiche, Diagnostiche e Pediatriche, Università degli Studi di Pavia, Pavia, Italy; 8 Dipartimento di Scienze Chirurgiche, Fondazione IRCCS Policlinico San Matteo, Pavia, Italy; 9 Wayne State University School of Medicine, Detroit, MI USA; 10 Jefferson College of Population Health, Philadelphia, PA USA; 11 Sigmund Freud University, Vienna, Austria

**Keywords:** Covid-19, Twitter, Non-pharmacological interventions, Social media, Communication

## Abstract

The COVID-19 pandemic changed expectations for information dissemination and use around the globe, challenging accepted models of communications, leadership, and social systems. We explore how social media discourse about COVID-19 in Italy was affected by the rapid spread of the virus, and how themes in postings changed with the adoption of social distancing measures and non-pharmaceutical interventions (NPI). We used topic modeling and social network analysis to highlight critical dimensions of conversations around COVID-19: 1) topics in social media postings about the Coronavirus; 2) the scope and reach of social networks; and 3) changes in social media content as the nation moved from partial to full social distancing. Twitter messages sent in Italy between February 11th and March 10th, 2020. 74,306 Tweets sent by institutions, news sources, elected officials, scientists and social media influencers. Messages were retweeted more than 1.2 million times globally. Non-parametric chi-square statistic with residual analysis to identify categories, chi-square test for linear trend, and Social Network Graphing. The first phase of the pandemic was dominated by social media influencers, followed by a focus on the economic consequences of the virus and placing blame on immigrants. As the crisis deepened, science-based themes began to predominate, with a focus on reducing the spread of the virus through physical distancing and business closures Our findings highlight the importance of messaging in social media in gaining the public’s trust and engagement during a pandemic. This requires credible scientific voices to garner public support for effective mitigation. Fighting the spread of an infectious disease goes hand in hand with stemming the dissemination of lies, bad science, and misdirection.

## Introduction

Since it emerged as a global threat in early 2020, the COVID-19 pandemic has affected health, human functioning and society on an unprecedented scale. The global spread of the virus in the absence of vaccines and effective treatments demonstrates the importance of effectively using non-pharmaceutical intervention (NPI) such as social distancing to reduce transmission of the virus, limit mortality and avoid overwhelming local healthcare systems [1]. Two strategies were used in most nations: quarantine of infected persons and social distancing to mitigate the spread of the virus [2–5]. Effective implementation of containment and social distancing strategies requires social trust, given the threat of massive disruption to society and the economy [6].

In response to the rapid spread of COVID-19, many nations mandated all but essential businesses to be shuttered and for individuals to “shelter in place” to reduce the risk of transmission of the highly contagious virus. In Italy, as one of the first countries to be severely hit by the wave, the “#I-stay-home” campaign obliged citizens to avoid leaving their homes. This effort and similar programs in other nations require trust and public consensus, to engage a nation’s citizens as active co-participants in their own and their fellow citizen’s health and well-being [7].

At the time of submission, almost 3 million cases of infection and nearly 100,000 COVID-19 related deaths had occurred in Italy. The effectiveness of measures such as social distancing to reduce the spread of the virus depends on the level of social trust and collection societal action that is supported by integration among the key groups such as citizens, institutions, information providers and elected officials [8]. Artificial dichotomies between the need to contain the spread of the virus and the need to maintain the health of the economy, conflicting themes in public and social media, and lack of a unified message can undermine the citizen buy-in, social trust, public compliance, and the speed and effectiveness of implementation.

Social trust and precise messaging are key in the current efforts to address an unprecedented challenge to the healthcare systems of nations. They are needed to inform public perceptions and contribute to a developing regional or national consensus that helps leaders and policymakers to coordinate transparent and consensus-based efforts to adopt of country-wide social distancing measures such as closing schools, banning mass gatherings, and isolating individuals with the virus and their contacts. These efforts were shown to be effective in containing the spread of the Spanish Flu in 1918 [9].

In this paper, we explore the content and messages in social media communications during the early stages of the spread of the COVID-19 virus in Italy, which numbers are reported in Appendix 1 (Table 4). The aim is to better understand how social media dialogue can affect and be used strategically in the adoption of large-scale regional and national social distancing measures to prevent the spread of the virus.

## Literature review

### NPI

The World Health Organization Influenza Pandemic Plan of 1999 puts considerable attention on the role of non-pharmaceutical public health interventions to contain or delay the spread of a new influenza virus [10]. NPI include early case isolation, social distancing using face masks, closing of schools and businesses shot-down [10]. The application of NPI proved to reduce the spread of the COVID-19 virus in several areas inside China [11–14]. However, to be effective, NPI requires authorities to agree in advance on a range of containment strategies, the population be informed and willing to adopt the necessary measures [10].

Analyzing the NPI applied during the influenza pandemic of 1918 Whitelaw [15] wrote: “To sum up, it is evident, that no public health law, which has not the endorsation and support of the public generally, can ever be reasonably well enforced.” More recently, the WHO [16] wrote: “Some of the lessons learned from the 2003 severe acute respiratory syndrome (SARS) epidemic can be applied to influenza, including the success of public campaigns to encourage self-recognition of illness, telephone hotlines providing medical advice, and early isolation when potential patients seek health care.” Several variables have proved necessary to get public endorsement for the application of NPI such as the perceived risk, severity of the consequences as well as response efficacy of the adopted measures [17, 18]. Therefore, while NPI has proved to be effective in limiting the spread of a pandemic, there must be a public endorsement of their employability. Giving people the right information is essential to empower them to evaluate their risks and the importance of curtailing their freedoms in terms of virus spread limitation.

### Emergency management and social media communication

The development of social media has changed the communication both in terms of information availability and flow. Collaborative generation and dissemination activities of several types of content are some of the most critical distinct features of social media. According to Brynielsson et al. [19] “Within the field of crisis communication, social media possibilities such as online sharing and social networking have had an impact on the way crisis information is disseminated and updated.” Among the many social media, Twitter has been widely used in emergency management literature due to its specific features. For example, Twitter allows to post comments visible to all audience but also directly targeting a specific audience due to the mention and reply function [20]. The hashtag feature might help support the rapid building of an issue around specific community problems or geographical areas [21].

Research on emergency management shows that Twitter has been used to improve situational awareness among communities [22]. It can inform local communities given emergency alerts [19], and can act as a tool to facilitate social and political trends for change during emergencies when emotions embolden people [23]. Despite its great potentiality, due to the unchecked and socially constructed nature, messages shared on Twitter might lead to disinformation contributing to the infodemic problem [24]. For example, Panagiotopoulos et al. [25] discuss the social amplification or reduction of risks that on the one hand might be caused due to the Twitter flow and that on the other hand could be monitored by those responsible for risk management. Similarly, Surian et al. [26] used Twitter discussions about human papillomavirus vaccines for clustering opinions and detecting risks for public health.

### The COVID-19 emergency and the Infodemic

The COVID-19 is a global emergency “which started in Wuhan in China in early December 2019, brought into the notice of the authorities in late December, early January 2020, and, after investigation, was declared as an emergency in the third week of January 2020” [27]. At the time we are writing the COVID-19 has killed almost 2.5 million people worldwide. However, just a few months earlier, the nature and danger of the virus were hotly contested. The US Surgeon General, Jerome Adams tweeted on February 1st, 2020 “Roses are red, violets are blue, risk is low for #coronarvirus, but high for the #flu” [28]. On March 9th, 2020 the US President Donald Trump tweeted: “Last year 37,000 Americans died from the common Flu. Nothing is shut down, life and the economy go on... Think about that” [28]. When it became clear that the situation was much worse, and commenting on his previous statements on Twitter he later said: “circumstances change but it was a true statement at the time it was made” [28]. Therefore, the COVID-19 emergency differs from other emergencies as knowledge of the real risks was mainly unknown or at least debated at the early stages of development of the pandemic.

The development of the COVID-19 pandemic demonstrates the spread of fake news, false information based on non-checked facts [29]. In March 2020, a pool developed by YouGov and the Economist revealed that 13% of Americans believed the COVID-19 crisis a hoax, while even world leaders’ social media posts had to be deleted for spreading misinformation about the Coronavirus [29, 30]. The development of false and unchecked information, recently named infodemic [24] during the COVID-19 emergency is peculiar compared to other crisis. The limited scientific knowledge available and the lack of developing consensus among the population increased the initial spread of the virus due to the specific nature of the NPI required.

Twitter has proved to be “the dominant social reporting tool to spread information on social crises” [31]. Previous studies employing crisis and emergency risk communication models are based on the monitoring of the risks and the communication of warnings [32] to avoid social amplification of the risks [33]. However, the COVID-19 emergency represents a new context, where little knowledge was available at the beginning of the crisis on the real dangers. Understanding how communications flow on Twitter, shaping the community understanding of the risks in a situation where there is little or debatable knowledge on the dangers appears, therefore, central.

## Methods

Our analysis included three steps. First, we explored the main topics in messages by five groups with regular twitter communication and sizable numbers of followers: institutions, news sources, elected officials, scientists and social media influencers using topic modelling methods. Second, we used social network analysis to assess the size and reach of social networks and identify boundary spanning opportunities (sources and messages that span social networks) [34]. Third, we conducted a chi-square trend analysis that analyzed the impact of the mounting crisis on the themes in social media message.

### Data collection

We downloaded tweets posted on the topic of COVID-19 infection in Italy from February 11th to March 10th, 2020. A tweet is an online posting created by a Twitter user limited to 280 characters or less. Once published, the tweet will appear on the Twitter home pages of all users who follow the induvial who released the message. Users might retweet messages, amplifying selected and extending the spread of certain discussions. Twitter is the most heavily used micro-blogging platform in the world and provides access to its data. Although Twitter represents only a part of available social media, a number of studies have used Twitter data, with studies showing it is a reasonable proxy and representation of political, social and scientific opinions [35, 36].

We selected tweets based on their contents using both keywords and the hashtags: virus, Coronavirus, and COVID-19. Other keywords, such as, for example, SARS-CoV-2, were excluded since the tweets mentioning those words were few and also reporting the word “virus”. We received messages tweeted in Italian from the Twitter company and focused on the top retweeted messages, using an inclusion criterion that included more than 50% of total retweeted messages and ignored messages that did not attract attention from users. We only used the number of retweets as a metrics of virality because, since our interest was about examining the infodemic phenomenon, we were interested in the diffusion of the messages, instead of considering the users’ reactions (e.g., likes, feelings, comments and replies).

### Data analysis

We analyzed the content in the data using Python (Python Software Foundation) and its topic modelling function to detect the main topics discussed in the messages using a computer-aided content analysis [37]. Content analysis provides a useful and multifaceted, methodological framework for Twitter analysis and supports the structuring of textual data by enabling categorizing and coding [38]. Within content analysis, topic modelling is a type of statistical modelling for discovering abstract “topics” that occur in a collection of documents or as in our case tweets. Latent Dirichlet Allocation (LDA) approach was used to classify and code text into particular topics [39].

The original list obtained from the statistical analysis was then manually coded by the authors (MM, PT, and MLT). The emerging codes were circulated among the researchers, and the list of codes was included in a codebook. Several conference calls/meetings were held to fine-tune the codebook and to group codes that related to the same phenomena. We further analyzed the data until conceptual saturation was reached and no new codes or categories were generated or merged together [40]. In addition, we manually coded the most retweeted messages by senders using the description provided by the users themselves in the presentation of their account using open coding [41]. In some cases, when the account’s presentation was not enough to define a sender, we searched his/her profession or role using the web. This coding approach means that we created new codes according to the senders’ descriptions of their accounts, so creating categories reflecting the concepts about the types of actors. We iterated the aggregation and creation of codes until reaching a conceptual saturation with significant categories of actors. Therefore, we aggregated senders of tweets into five distinct categories: *Institutions* (e.g., messages from the government or the Italian NHI), *News* sources(e.g., messages from TV channels or journalists), *Politicians* (e.g., messages from personal accounts of politicians or political parties), *Science Sources* (e.g., messages from scientists), and *Influencers* (i.e. all the other influencing users, including V.I.P.’s, celebrities, and private users who accounted for a large number of retweets, using a cut-off point of 1400 retweets).

We employed a chi-square **test of independence** with standardized residuals to search for similarities and differences in topics discussed by source (e.g. topics mainly discussed by institutions, politicians, etc.) using R software [42].

As a second step, we analyzed the development of discussions and messages over time. We chose three periods: a) before February 24th; from Feb 25th to March 1st, when the number of infected individuals exceeded 200, and few regions of the country had implemented social distancing; and c) between March 1st and March 10th when the entire country was in lock-down. The numbers related to the daily spread of the disease are reported in Appendix 1 (Table 4). The social network map using the ForceAtlas2 algorithm [43] was produced using the software Gephi, open-source software for graph and network analysis that measures the relationships and flows between people, groups, or organizations [44, 45]. The layout provided by the software supported the grouping and alignment of nodes connected together and helped to determine the current community state of social networks and to identify boundary spanning opportunities.

As a third step, a chi-square trend analysis was employed to search for linear trends between the COVID-19 crisis and the number of retweets from each source (i.e., influencers, institutions, news, politicians, scientific sources) for each topic and the total number of retweets were analyzed and compared to available COVID-19 morbidity and mortality data.

## Results

### Topic analysis

Our data encompassed 74,306 messages that were retweeted more than 1.2 million times from a total number of 2.3 million assessed retweets. The data analysis revealed 14 major themes that were intensely discussed by the five groups. Table 1 reports the main topics discussed, with examples of each type of message, the number of retweets and the keywords used in the classification process. The chi-square analysis revealed significant differences between the topics discussed by each group (χ^2=^8437.5, df = 52, *p* < 0.001). We produced a double-entry table (topics of rows and actors on columns) and compared the actual results with expected results from the chi-square analysis. The differences between the expected versus the actual results were then divided by the square root of the variance function/expected value to obtain the Pearson’s residuals.

**Table 1:** Themes, Categories, Codes, and Quotes from Most Retweeted Messages

**Topic Label**	**Keywords**	**Examples (Highest retweets)**
Fear of foreigners’ reaction	Africa, Italy, Italian, migrants, quarantine, ports, government, illegal immigrants, NGOs, Chinese people, fear, to close, after, case, first	I have never been a defeatist, but it is difficult not to become one. There are too many ignorant, arrogant and selfish people in our country. The vaccine issue had suggested it, coronavirus confirms it dramatically.
	China, Chinese people, [politician X], Tuscany, quarantine, state, governor, [politician W], [politician Z], [politician Y], declaration, president, to be, risk	“Are you worried about Coronavirus?” People from Veneto region: “we have the alcohol that protects us” People from Tuscany: “I don’t give a damn” People from Bari: “I have not taken anything, neither masks nor anything, I want to die” I have tears in my eyes.
Infection risk and epidemic information	Italy, what, to be, can, contagion, only, WHO, epidemic, China, covid19italy, diffusion, people, days, situation, need	“You make unprotected sex and you are afraid of Coronavirus contagion”
	Sacco [hospital], Milan, hospital, director, today, first, vaccine, emergency, Italian, [virologist 1], epidemic, service, interview, I follow the news, work	She swapped an infection just more serious than flu for a lethal pandemic. It is not so.
Closing schools and universities and sports competitions	schools, closed, March, Milan, closing, Universities, Italy, all, Lombardy, Rome, region, government, closed, closes, Veneto	“Coronavirus, schools and universities closed forever.”
	Serie A League, no public, football, Italy, Minister, Sports, Health, postponed, hope, update, stop, emergency, games, championship, first	#coronavirusitalia, soccer matches with closed doors for 12 Serie A teams: Juventus, Turin, Inter, Milan, Atalanta, Brescia, Verona, Udinese, Bologna, Parma, Sassuolo and Spal. Volleyball and rugby also stop
Regional restrictions to mobility	Lombardy region, government, Italy, decree, quarantine, red zone/lockdown zone, [politician C], Veneto, measures, [politician Y], checks, regions, Milan, all	“Venice: free aperitif in St. Mark’s Square to start over again.” But did you understand that people must stay at home otherwise what starts again is the virus?
	home, Milan, I stay at home, fear, Italy red zone, Rome, supermarkets, mayor, what, networking, appeal, state, rules, quarantine, people	#Istayathome Leave the virus out the door. Stay home and go out only for essential needs.
Early cases of infection and risk of infection	cases, positive, two, patients, hospital, Lombardy, test, intensive care, region, people, Veneto, Spallanzani [hospital], hospitalized, emergency, Rome	While everyone was looking at poor immigrants arriving by boat and waiters at Chinese restaurants, the virus travelled comfortably in first class with a manager from Lombardy #coronavirus
	case, first, positive, years, hospital, patient, infected, woman, state, man, hospitalized, Lombardy, Italy, test	My mom works in the ER of #codogno where this gentleman was [Patient 1]. You don’t know how much it hurts to know that she and all her colleagues will be quarantined for 15 days. Those who do this work should be thanked every day for what they do
	China, quarantine, Italy, Italian, WHO, Wuhan, passengers, people, ship, Chinese, Diamond_Princess, Italians, epidemic, dead	ER of Rimini. 81 years old. Cough, Coronavirus-positive There is no place for him because he is over 80.If he heals, it will be because he did it alone.You pay taxes for a lifetime, and that’s what comes back.Now tell me .... What would you do if you were one of his children or grandchildren and he died?
Economic crisis and economic support to people and business	Italy, economy, crisis, EU, billions, emergency, China, tourism, GDP, recession, Milan, today, stock market, decline, Europe	Paranoia coronavirus, there are those who launch SOS to central banks: Fed and ECB intervene with concerted action to save the economy (or the markets?) Worst week since 2008, global stock burns $ 5 trillion. Record for fear index
	measures, emergency, government, decree, count, Italy, businesses, economy, work, all, health, billions, new, minister, companies	Today meeting a pharmacist. Me: “How do you see the situation?” He: “if we had sold the equivalent of today’s masks in condoms 40–50-60 years ago, today we would have had fewer assholes on social media that talk about viruses as experts.”
Updates infections and trends in Italy	cases, Italy, dead, update, contagions, infected, Lombardy, covid19italy, China, updates, deaths, new, hours, number, coronarvirusitaly	“However guys, we have all the countries with the highest number of coronaviruses in the world: Korea, China and Italy”
Risk of death for vulnerable people	flu, years, only, dead, elderly, first, people, dies, dead, die, sick, pathologies, Italy, say, what	I meet my nieghbour over eighty years old. I ask her if she is worried, answer “here you all think about the virus, but there are 18 degrees at the end of February when there should be 2, you all seem stupid”.Ok.
Propaganda, conspiracy and polemic	only, then, Italy, what, made, people, world, to be, fear, first, country, problem, Italians, say, government	A tyrant has turned our lives upside down, and it’s called coronavirus. We will resist and fight everywhere, in our homes, in the workplace. Helping the weakest and sacrificing ourselves for a better tomorrow. And then we’ll do it again. Coronavirus, you won’t win. We hunted worse.
Criticism of government’s action	[politician C], government, [politician Z], emergency, Italy, made, country, only, league, premier, [blogger], [politician V], [Institution A], Italians,	In my opinion, Cosimo de ‘Medici [historical character] managed the plague in Florence much better than how we are managing the coronavirus these days.
Information on coronavirus around the world	Italy, Germany, France, cases, USA, Europe, masks, first state, emergency, EU, China, countries, outbreak, country	#China is the only country that supports Italy: it donated 1000 lung ventilators, 50,000 # coronavirus swabs, 20,000 protective suits, 100,000 high-tech masks and 2 million ordinary medical masks.
Information and instructions for people behaviour	video, contagion, pizza, news, information, tv, photo, social, covid19italY, fake_news, rai [broadcaster], rules, Italy, only, Ansa [news]	For exceptional moments we need exceptional behaviour. Stay home and follow the directions of the Ministry of Health.
European Union’s role and action for COVID emergency	Italy, Eu, Europe, ESM, migrants, government, money, emergency, health, state, country, years, only, billions, Greece	“Anyway, the EU has not yet taken any significant initiative against the coronavirus epidemy, except for authorizing us to spend money coming from our debts. So, it did only one thing: wasting our time asking for the authorization.”
Hospitals emergency	emergency, doctors, hospitals, masks, Italy, Lombardy, first, healthcare, [Institution A], president, medical doctors and nurses, country, millions, great, moment	#coronavirus: statement by the President #Mattarella

**Fig. 1:**
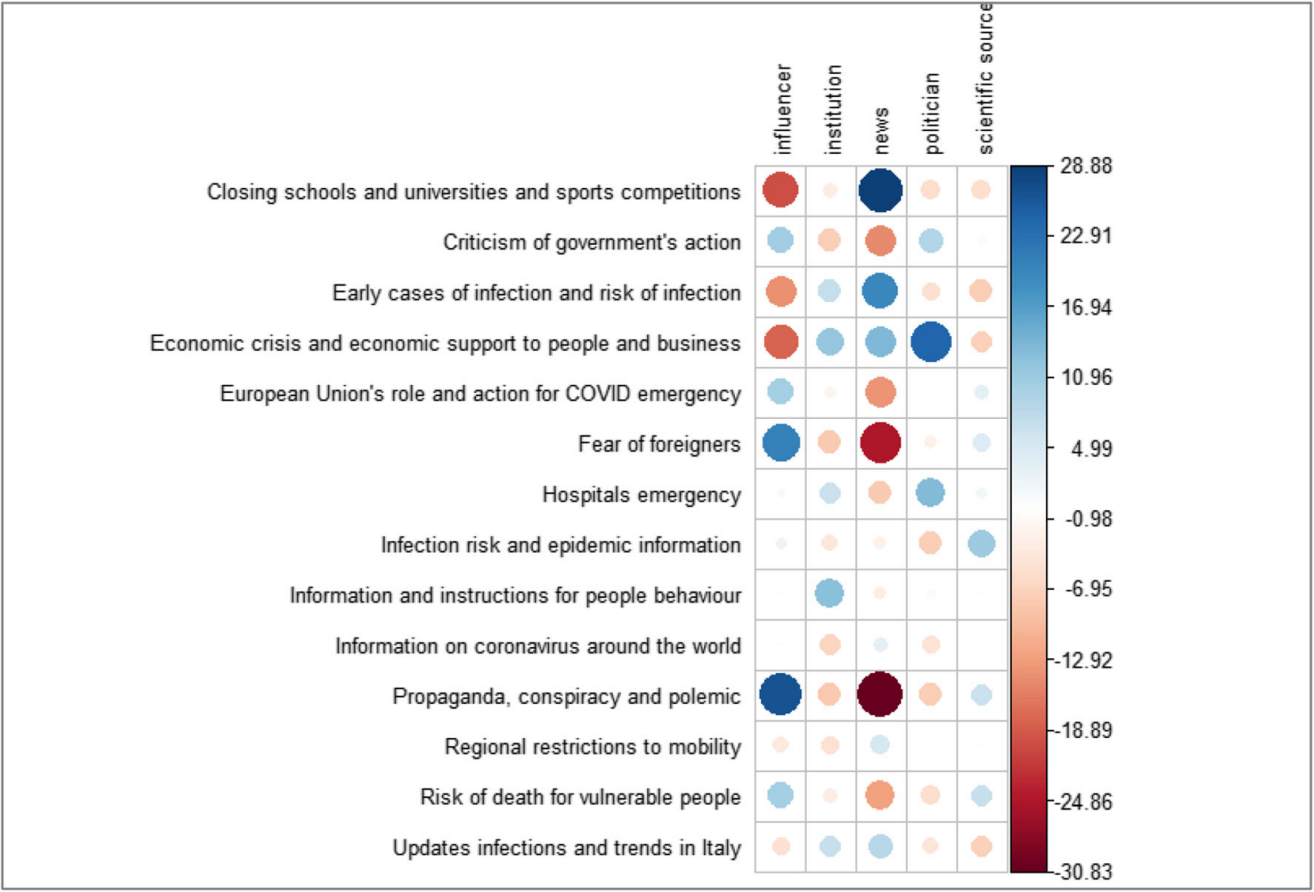
Topic Analysis by Actor Type

The topic analysis was developed using the function chisq in R. The results are shown in Fig. 1. Positive residuals are coloured in blue, defining an attraction between the corresponding rows (topics) and the column (actors). Negative residuals are coloured in red showing repulsion (negative association) between the corresponding row and column variables. The results show that influencers had higher standardized residuals, suggesting a higher than expected number of messages for the specific actor for messages that spoke to fear of foreigners and blamed immigrants in Italy for starting the COVID-19 outbreak. Politicians had higher residuals (suggesting higher than expected numbers of messages) for messages connected with managing the economic fallout, and to support citizens and businesses and hospital during the crisis. Not surprisingly, infection risks and rates and epidemiological information commonly originated from Scientific sources. In contrast, News sources were mainly concerned with the closing of entertainment, restaurants, schools and universities, identifying early cases of infections and highlighting the slowdown in the economy. The Institutional sources had a higher propensity for information and guidance for directing the behaviour of citizen.

### Actor type relevance

**Fig. 2:**
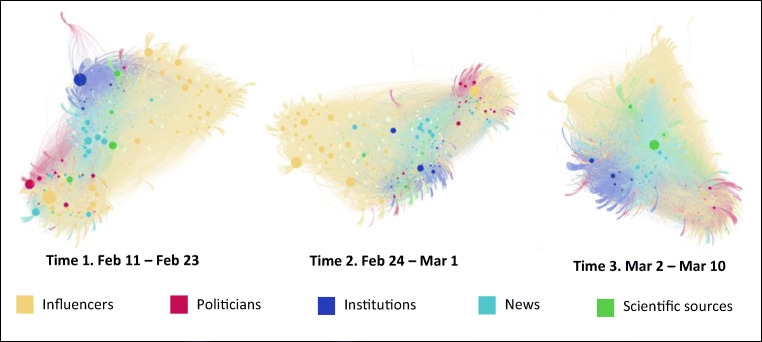
A Social Network Graph Based on Tweets

The findings for the Social Network Analysis suggested a prevalence of specific messages during the three periods. (Fig. 2) During the first time-period, messaging was dominated by influencers with several prominent actors that attracted and guided the national discourse. The results demonstrated, however, that during the most critical days, February 19th and 20th, when the first Italians tested positive for COVID-19 [46], the average percentage of retweets for influencers fell from 55% to 25% of the daily total, with scientific sources rising from an average of 8% to 42–48% of total tweets. The second time-period shows that the news channels and broadcasters were receiving more attention, but influencers were still relevant and often undermined the scientific messaging. During the third time-period, scientific sources began to dominate the discussion, building public confidence with messaging flow that was topically congruent and connected to news sources with both emphasizing key messages for dealing with the pandemic.

### Topic and actor type relevance during the crisis development

The chi-square trend analyses follow the three main periods previously discussed (Tables 2 and 3). During the three time-periods, the infection rates were increasing with the total number of cases moving from three cases on Feb 11th to 1694 cases on March 1st, and to 10,142 cases on March 10th. The results showed that some topics dropped dramatically in their trending as the crisis intensified. This includes tweets promoting anti-immigrant propaganda and fearmongering against foreigners. Other topics, particularly science-based and practical information, grew in their relative importance and urgency. The reduced messaging by influencers made room for a range of sources to contribute solutions and build confidence. This included scientific sources, politicians and institution, who collectively contributed to messaging that sought to build social trust and community activation.

**Table 2:** Chi Square Trend Analysis of the Key Topics in the Three Time Periods

**Topic**	**Feb 11 – Feb 23**	**%**	**Feb 24 – Mar 1**	**%**	**Mar 2 – Mar 10**	**%**	χ^2^ **Trend**		***p*** **value**
Closing schools and universities and sports competitions	15,961	5.4%	24,511	7.1%	46,060	8.0%	↑	2028.10	<0.001
Criticism of government’s actions	23,169	7.8%	31,132	9.0%	30,936	5.4%	↓	2576.70	<0.001
Early cases of infection and risk of infection	66,651	22.4%	46,173	13.3%	54,190	9.4%	↓	26,666.0	<0.001
Economic crisis and economic support to people and business	11,838	4.0%	26,327	7.6%	46,260	8.1%	↑	4434.10	<0.001
European Union’s role and action for COVID emergency	4972	1.7%	8044	2.3%	25,682	4.5%	↑	5698.40	<0.001
Fear of foreigners	40,587	13.7%	40,163	11.6%	27,264	4.7%	↓	22,020.0	<0.001
Hospitals emergency	5610	1.9%	13,550	3.9%	50,103	8.7%	↑	19,044.0	<0.001
Infection risk and epidemic information	41,526	14.0%	32,952	9.5%	57,651	10.0%	↓	2493.40	<0.001
Information and instructions for people behaviour	10,095	3.4%	18,302	5.3%	23,418	4.1%	–	70.79	<0.001
Information on coronavirus around the world	7805	2.6%	17,907	5.2%	33,962	5.9%	↑	4198.70	<0.001
Propaganda	30,961	10.4%	30,338	8.7%	49,117	8.6%	↓	732.18	<0.001
Regional restrictions to mobility	17,663	5.9%	19,352	5.6%	88,029	15.3%	↑	23,588.0	<0.001
Risk of death for vulnerable people	7697	2.6%	20,138	5.8%	19,926	3.5%	–	60.35	<0.001
Updates on infections and trends in Italy	12,396	4.2%	18,371	5.3%	21,763	3.8%	–	201.63	<0.001
**Total**	**296,931**	**100.0%**	**347,260**	**100.0%**	**574,361**	**100.0%**

**Table 3:** Chi Square Trend Analysis of the Key Actors in the Three Time Periods

**Actors**	**Feb 11 – Feb 23**	**%**	**Feb 24 – Mar 1**	**%**	**Mar 2 – Mar 10**	**%**	χ^2^ **Trend**		**p value**
Influencer	173,829	58.5%	191,853	55.2%	269,989	47.0%	↓	11,696.0	<0.001
Institution	13,616	4.6%	19,368	5.6%	46,727	8.1%	↑	4531.80	<0.001
News	64,147	21.6%	80,663	23.2%	155,994	27.2%	↑	3632.70	<0.001
Politician	27,650	9.3%	40,907	11.8%	49,330	8.6%	–	376.97	<0.001
Scientific source	17,689	6.0%	14,469	4.2%	52,321	9.1%	↑	4547.50	<0.001
**Total**	**296,931**	**100.0%**	**347,260**	**100.0%**	**574,361**	**100.0%**			

## Discussion

This paper contributes to the significant body of literature examining the COVID-19 pandemic. Our results show the importance of social media in supporting a community-wide and ultimately nationally-coordinated effort to build public awareness and engagement during the COVID-19 pandemic. Analysis of social media themes highlights useful and damaging messages, including false claims that blamed COVID-19 on foreigners. Interestingly, several actors without a scientific background encouraged discussions about how best to prepare for COVID-19. In contrast, contentious and dissenting voices might slow the process to reach a fact-driven consensus, and even promote counterproductive actions such as downplaying the danger of public crowding.

Twitter and other social and digital means of communication have become essential channels for physicians and scientists to spreading health and public health information [47, 48]. Twitter proved to be a powerful knowledge translation tool to translate and transfer meaningful knowledge from healthcare authorities to the population, about what should be done or avoided [49, 50]. Twitter feed ultimately benefitted the global community during the pandemic by serving as a readily accessible and trusted source for reliable and science-based information [48]. COVID-19 also highlighted the danger of a serious infodemic [51], with an over-abundance of information with uncertain accuracy, making it difficult for individuals to select the sources for actionable information and guidance.

Political actions and actors impacted the coronavirus spread, by denying the COVID-19 realities, and promoted social interactions under the motto “let’s keep our habits, we can’t stop Milan and Italy.” These public actions effectively helped spread the virus, only to back-track days later as the number of affected COVID-19 people dramatically increased and the pandemic mortality emerged [52]. Some tweets contained messages promoting fear and falsely blaming foreigners’ for the illness and created a false promotion that underestimated the severe impact of COVID-19. These actions undermined social trust and preparedness, exacerbating a general sense of fear and panic across Italy as the pandemic was fast becoming a debilitating national emergency.

There are many lessons to be learned for other nations about the experience of social media messages in Italy. Paramount is the importance of clear, convincing, fact-based and actionable messaging, to overcome misinformation and to garner the trust of the public during a challenging time. A handful of countries, including Singapore, Taiwan, Germany, Iceland, have managed to stay on top of their outbreaks by adhering to radical transparency, promoting community activism, while aggressively testing to find cases, quarantining contacts, and keeping viral transmission from going into an exponential growth phase [53]. Taiwan’s early recognition of the crisis, daily briefings to the public, and simple health messaging allowed the government to reassure the public by delivering timely, accurate, and transparent information regarding the evolving epidemic [54]. Additionally, the lessons from China and Singapore show that when the NPI reached the target of limiting the Covid-19 spread, new emerging cases can require the reintroduction of containment measures. Timeliness in accurate public messaging using both smart technology and traditional press conferences by trusted leaders was crucial.

Our study has inherent limitations. While Twitter has become an essential platform for textual communication and information sharing, the tweets represent only a sample of people’s communication and human interactions. However, there are many studies using Twitter data that consider it a reasonable proxy of the user’s mental models [42, 55]. We realize that the tweets also exhibit specific characteristics of brevity, fluidity, and meaning embedded in a broader context. This can pose challenges for the researcher engaged in content analyses. Third, the systematic limitation to analyze twitter methods “statistically” may be appropriate but as of yet un-validated for analyzing twitter, key messages, key actors, and evolution over time. Fourth, the COVID-19 pandemic and its rapidity evolving social, psychological, economic, or geographically attributes mean that the analysis is accurate only in the context of the limited time frame being studied. The co-variates may undoubtedly alter the findings and statistical results to where they are applied. Further development of statistical procedures which can be validated and replicated is needed with this type of data. Fifth, our analysis focused only on tweets posted in Italy. The different spread of social networks among generations or genders in different cultures and countries might affect our results. Finally, we focused on a limited period of time. Extending our analyses likely could have led to different findings.

## Conclusions

Societies need to respond quickly to pandemics to protect the health and well-being of their citizens. In this exploratory work, we developed a systematic method to analyze Twitter messages, understand key messages, key actors and their evolution over time. We showed that social media could be used effectively to respond to the pandemic through transparent and convincing messages, rooted in scientific knowledge, to help build confidence and improve the implementation effectiveness of policies, ending up as an effective knowledge translation tool to facilitate the communication with the population. Despite the infodemic, the threats from fake news, trolls and bots that automatically produce and share contents on social media, the scientific voice and other institutional sources of information were able to dominate and be spread among people over the acute phase of the outbreak, so gaining their trust and public engagement in facing the pandemic. Countries that are transparent on the state of their country and provide truthful health information for their citizens will likely gain public trust and more rapid NPI uptake and compliance. Finally, we believe that an area for future research entails examining how social media and other readily collected public data could be leveraged to improve methods for public messaging, assessing the spread of the virus and support appropriate public health actions. Twitter can be leveraged to improve population health preparedness, better and early public response and support public policy actions [56].

## Appendix 1

**Table 4:** Daily progression of the disease in Italy

**Date**	**Country**	**Hospitalization (not in ICU)**	**ICU occupation**	**Total Hospitalized**	**Self-isolated**	**People Tested Positive (total)**	**People Tested Positive (per day)**	**People recovered**	**Number of deaths**	**Total cases**	**People tested (total)**
2/24/2020	ITA	101	26	127	94	221	221	1	7	229	4324
2/25/2020	ITA	114	35	150	162	311	90	1	10	322	8623
2/26/2020	ITA	128	36	164	221	385	74	3	12	400	9587
2/27/2020	ITA	248	56	304	284	588	203	45	17	650	12,014
2/28/2020	ITA	345	64	409	412	821	233	46	21	888	15,695
2/29/2020	ITA	401	105	506	543	1049	228	50	29	1128	18,661
3/1/2020	ITA	639	140	779	798	1577	528	83	34	1694	21,127
3/2/2020	ITA	742	166	908	927	1835	258	149	52	2036	23,345
3/3/2020	ITA	1034	229	1263	1000	2263	428	160	79	2502	25,856
3/4/2020	ITA	1346	295	1641	1065	2706	443	276	107	3089	29,837
3/5/2020	ITA	1790	351	2141	1155	3296	590	414	148	3858	32,362
3/6/2020	ITA	2394	462	2856	1060	3916	620	523	197	4636	36,359
3/7/2020	ITA	2651	567	3218	1843	5061	1145	589	233	5883	42,062
3/8/2020	ITA	3557	650	4207	2180	6387	1326	622	366	7375	49,937
3/9/2020	ITA	4316	733	5049	2936	7985	1598	724	463	9172	53,826
3/10/2020	ITA	5038	877	5915	2599	8514	529	1004	631	10,149	60,761
3/11/2020	ITA	5838	1028	6866	3724	10,590	2076	1045	827	12,462	73,154
3/12/2020	ITA	6650	1153	7803	5036	12,839	2249	1258	1016	15,113	86,011
3/13/2020	ITA	7426	1328	8754	6201	14,955	2116	1439	1266	17,660	97,488
3/14/2020	ITA	8372	1518	9890	7860	17,750	2795	1966	1441	21,157	109,170
